# Molecular characterization of a long range haplotype affecting protein yield and mastitis susceptibility in Norwegian Red cattle

**DOI:** 10.1186/1471-2156-12-70

**Published:** 2011-08-11

**Authors:** Marte Sodeland, Harald Grove, Matthew Kent, Simon Taylor, Morten Svendsen, Ben J Hayes, Sigbjørn Lien

**Affiliations:** 1 Centre for Integrative Genetics, Department of Animal and Aquacultural Sciences, Norwegian University of Life Sciences, N-1432 Aas, Norway; 2 Geno Breeding and AI organization, Norwegian University of Life Sciences, Box 5003, N-1432 Aas, Norway; 3 Biosciences Research Division, Department of Primary Industries Victoria, Melbourne, Australia, 3083

## Abstract

**Background:**

Previous fine mapping studies in Norwegian Red cattle (NRC) in the region 86-90.4 Mb on *Bos taurus *chromosome 6 (BTA6) has revealed a quantitative trait locus (QTL) for protein yield (PY) around 88 Mb and a QTL for clinical mastitis (CM) around 90 Mb. The close proximity of these QTLs may partly explain the unfavorable genetic correlation between these two traits in NRC. A long range haplotype covering this region was introduced into the NRC population through the importation of a Holstein-Friesian bull (1606 Frasse) from Sweden in the 1970s. It has been suggested that this haplotype has a favorable effect on milk protein content but an unfavorable effect on mastitis susceptibility. Selective breeding for milk production traits is likely to have increased the frequency of this haplotype in the NRC population.

**Results:**

Association mapping for PY and CM in NRC was performed using genotypes from 556 SNPs throughout the region 86-97 Mb on BTA6 and daughter-yield-deviations (DYDs) from 2601 bulls made available from the Norwegian dairy herd recording system. Highest test scores for PY were found for single-nucleotide polymorphisms (SNPs) within and surrounding the genes *CSN2 *and *CSN1S2*, coding for the β-casein and α_S2_-casein proteins. High coverage re-sequencing by high throughput sequencing technology enabled molecular characterization of a long range haplotype from 1606 Frasse encompassing these two genes. Haplotype analysis of a large number of descendants from this bull indicated that the haplotype was not markedly disrupted by recombination in this region. The haplotype was associated with both increased milk protein content and increased susceptibility to mastitis, which might explain parts of the observed genetic correlation between PY and CM in NRC. Plausible causal polymorphisms affecting PY were detected in the promoter region and in the 5'-flanking UTR of *CSN1S2*. These polymorphisms could affect transcription or translation of *CSN1S2 *and thereby affect the amount of α_S2_-casein in milk.

Highest test scores for CM were found in the region 89-91 Mb on BTA6, very close to a cluster of genes coding for CXC chemokines. Expression levels of some of these CXC chemokines have previously been shown to increase in bovine mammary gland cell lines after exposure to bacterial cell wall components.

**Conclusion:**

Molecular characterization of the long range haplotype from the Holstein-Friesian bull 1606 Frasse, imported into NRC in the 1970s, revealed polymorphisms that could affect transcription or translation of the casein gene *CSN1S2*. Sires with this haplotype had daughters with significantly elevated milk protein content and selection for milk production traits is likely to have increased the frequency of this haplotype in the NRC population. The haplotype was also associated with increased mastitis susceptibility, which might explain parts of the genetic correlation between PY and CM in NRC.

## Background

An unfavourable genetic correlation between PY and clinical mastitis (CM) has been reported in Norwegian Red cattle (NRC), with estimates ranging from 0.21 to 0.55 [1]. This genetic correlation could be both due to pleiotropic effects and to QTLs affecting the two traits being closely positioned on bovine chromosomes. The heritability for PY is estimated to be 0.19 in NRC [1], and is higher than the heritability for CM for which estimates range between 0.02 and 0.12 in Nordic cattle populations [2-4]. CM is inflammation of the mammary gland and the most costly disease affecting dairy cattle world-wide. In addition to economical considerations the disease also affects animal welfare. Milk production traits were included in the breeding goal for NRC earlier than health traits were, and selective breeding for PY could have increased the frequency of variants with undesirable effects on CM.

It has been suggested by Lien *et al*. [5] that a haplotype encompassing the casein gene cluster around 88 Mb on *Bos taurus *chromosome 6 (BTA6), which confers a favorable effect on milk production traits, was introduced into the NRC population through the importation of a Swedish Holstein-Friesian bull (1606 Frasse) in the 1970s. This bull has had a major influence on NRC, with an estimated contribution to this population above 8% (Svendsen, personal communication). Frasse 1606 and his sons were favored due to high breeding values for milk production traits before animal health traits were included in NRC breeding schemes, and might have contributed to generating a positive correlation between PY and CM in this population.

Association mapping has revealed that a quantitative trait locus (QTL) for protein yield (PY) coincides with the casein gene cluster [6-10]. Casein proteins constitute approximately 80% of dairy cattle milk protein and polymorphisms in these genes have been shown to contain variation associated with milk protein composition and protein content in other populations [5,7,11-16]. It has further been reported that the haplotype from 1606 Frasse encompassing the casein gene cluster is associated with increased mastitis susceptibility, and a QTL for CM in the periparturient period has been found around 90 Mb, close to the QTL affecting PY around 88 Mb [6]. Taken together these results suggest that causal polymorphism(s) residing within this genomic region may be influencing these two important traits.

Opportunities for fine mapping and molecular characterization of QTL regions have been improved by recent developments in high throughput sequencing and genotyping technologies [17-21], and it is well established that genotyping of related animals with well documented pedigree increases the accuracy of haplotyping and imputation methods [22,23]. Accuracy of imputation and power of association mapping using imputed genotypes is increased in populations with extensive linkage disequilibrium (LD) [24-27], meaning that a combination of these approaches is a feasible strategy in cattle populations [28-30].

Aims of this study were to refine the location of causative polymorphisms on BTA6 affecting PY and CM, and perform molecular characterization of the 1606 Frasse haplotype by re-sequencing in order to identify the most plausible causative polymorphism(s) underlying the two QTLs.

## Results and discussion

Re-sequencing in the genomic region between 86 and 97 Mb on BTA6 was done by first capturing sequence from seven genomic DNA samples using a Nimblegen sequence capture array, and then sequencing the product on a Roche 454 GS-FLX sequencer [19]. The seven samples included four NRC sires and three pools of old Norwegian breeds. Sequence data was aligned to the BTA_4.0 reference genome [31], using the MOSAIK software package and standard alignment parameters [32], and SNPs detection was performed with GigaBayes [33]. A total of 269 new single-nucleotide polymorphisms (SNPs) were revealed in this region and were genotyped in 768 NRC sires. The resulting dataset was joined with datasets containing additional SNPs previously genotyped in NRC by haplotyping and imputation of untyped genotypes. Imputation was facilitated both by the elevated LD in NRC and by extensive pedigree records being available [28-30,34,35]. Pedigree records improve haplotyping accuracy and thereby improve accuracy of association mapping [22,23]. The final imputed dataset contained genotypes for 556 SNPs in 2601 NRC sires, with typically only 1.2% of SNPs missing for each individual sire. Average distance between adjacent markers for the 556 SNPs included in this study was approximately 20 kb, with some variation in SNP density across the 11 Mb genomic region. The 556 SNPs are presented in Additional file 1 and pair-wise LD between these SNPs are shown in Additional file 2.

### Association mapping

**Figure 1:**
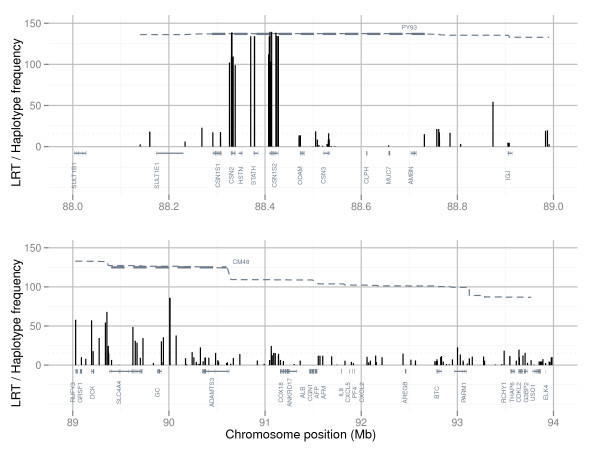
**Single-marker association mapping for protein yield**. Likelihood-ratio test (LRT) scores from single-marker association mapping for protein yield (black) in the interval 88 to 89 Mb (top) and 89 to 94 Mb (bottom) on Bos taurus chromosome 6 (BTA6). Significance threshold for LRT (>15.42) is indicated (black dotted line). Frequencies (frequency·1000) of the 1606 Frasse haplotype (grey thin dotted line) extending from the PY93 haplotype window, as well as haplotype windows PY93 and CM48 (grey thick dotted lines), are shown.

**Figure 2:**
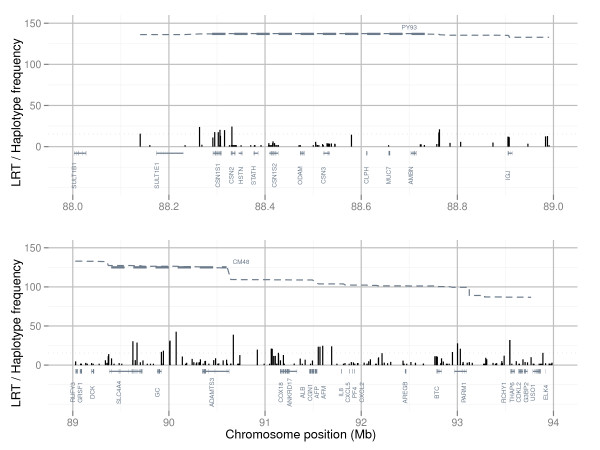
**Single-marker association mapping for clinical mastitis**. Likelihood-ratio test (LRT) scores from single-marker association mapping for clinical mastitis in the periparturient period of first lactation (black) in the interval 88 to 89 Mb (top) and 89 to 94 Mb (bottom) on Bos taurus chromosome 6 (BTA6). Significance threshold for LRT (>15.42) is indicated (black dotted line). Frequencies (frequency ·1000) of the 1606 Frasse haplotype (grey thin dotted line) extending from the PY93 haplotype window, as well as haplotype windows PY93 and CM48 (grey thick dotted lines), are shown.

Association mapping was performed to map single SNPs and haplotypes associated with PY or CM in the genomic interval between 86 and 97 Mb on BTA6. Three mastitis traits were included in the analyses; incidences of CM in the periparturient period of first (CM1), second (CM2), and third lactation (CM3). Haplotype blocks were defined by the algorithm developed by Gabriel *et al*. [36] (GAB) and by the four-gamete rule algorithm (GAM) described by Wang *et al*. [37], and all haplotype blocks defined by the GAB or the GAM algorithm were included in haplotype association mapping. Results for single-marker association mapping for CM1 and PY for the highest scoring region 88 to 94 Mb are shown in Figure 1 and Figure 2, whereas single SNPs and haplotype blocks giving highest test scores for each of the four traits are presented in Table 1.

**Table 1:** Association mapping results

Trait	Analysis	Position (bp)	LRT score	SNPs
CM1	SM	90,075,263	42	rs42766480
CM1	GAB	90,670,190 - 90,725,368	37	ss61522200 to ss290490303
CM1	GAM	90,642,598 - 90,670,190	42	rs29024027 to ss61522200
				
CM2	SM	89,625,793	26	ss290490281
CM2	GAB	89,623,896 - 89,625,793	23	ss61524338 to ss290490281
CM2	GAM	89,623,896 - 89,668,440	28	ss61524338 to ss86278591
				
CM3	SM	90,075,263	26	rs42766480
CM3	GAB	90,670,190 - 90,725,368	18	ss61522200 to ss290490303
CM3	GAM	90,642,598 - 90,670,190	19	rs29024027 to ss61522200
				
PY	SM	88,410,501	139	ss86217862
PY	SM	88,413,712	139	ss86217864
PY	GAB	88,333,706 - 88,422,590	78	ss86217849 to ss86217869
PY	GAM	88,333,706 - 88,427,761	78	ss86217849 to ss117968525

Highest test scores from single-marker (SM) and haplotype (GAB and GAM) association mapping for protein yield (PY) and clinical mastitis in the periparturient period of first (CM1), second (CM2) and third lactation (CM3). Trait, analysis, chromosome position, Likelihood-ratio test (LRT) score and SNPs are presented for the highest scoring SNP or haplotype block from each analysis.

Highest likelihood-ratio test (LRT) scores for PY were found for the SNPs ss86217862 and ss86217864 located at positions 88.410 Mb and 88.414 Mb. Both SNPs were positioned within the gene *CSN1S2 *and in complete LD with each other (r^2 ^= 1). The most significant haplotype results for PY were detected for a GAM block in the interval 88.33 to 88.43 Mb and a smaller GAB block lying within this interval (88.33 to 88.42 Mb). Both blocks encompassed the genes *CSN2*, *HSTN*, *STATH *and *CSN1S2*. Haplotype analyses for these two blocks did not reveal genome-wide significant test scores for any of the three mastitis traits.

Single-marker association mapping for CM1 and CM3 gave highest test scores for SNP rs42766480 at 90.07 Mb, whereas highest test score from haplotype association mapping were found for a three-marker GAM block in the interval 90.64 to 90.67 Mb. In contrast to CM1 and CM3, single-marker association mapping for CM2 gave highest test scores for SNP ss290490281 at 89.63 Mb and the most significant haplotype association for CM2 was for a four-marker GAM block in the interval 89.62 to 89.67 Mb. For all three mastitis traits highest test scores from both single-marker and haplotype association mapping were found within the interval 89 to 91 Mb. The SNPs that gave highest test scores for the three mastitis traits (rs42766480 and ss290490281) also gave high test scores for PY (Figure 1 and Figure 2). However, the SNPs rs42766480 and ss290490281 were not in high LD, as measured by r^2^, with the two SNPs that gave the highest test scores for PY in this study (r^2^_rs42766480,ss86217862/ss86217864 _= 0.091, D'_rs42766480,ss86217862/ss86217864 _= 0.734, r^2 ^_ss290490281,ss86217862/ss86217864 _= 0.046 and D'_ss290490281,ss86217862/ss86217864 _= 0.361). The latter indicates that the two traits PY and CM are affected by different QTLs.

### Molecular characterization of a long range haplotype affecting protein yield

Very high LRT scores for PY were found for SNPs within and surrounding the casein genes *CSN2 *and *CSN1S2 *(Figure 1). A previous study using the same population also identified this region and postulated that a influential haplotype associated with elevated protein yield was introduced into NRC through importation of the bull 1606 Frasse in the 1970s [7]. To identify possible causal polymorphisms underlying this QTL whole genome re-sequencing was conducted of five elite sires in the NRC population including 1606 Frasse (10× coverage) and two of his sons; 1893 Rud and 2005 Smidesang (both at 4× coverage).

A comparison of the sequence data covering the genes *CSN2 *and *CSN1S2 *and their 2000 bp 5'-flanking promoters with 28 previously genotyped SNPs within these regions showed that only one SNP out of the 28 SNPs was undetected by the re-sequencing. Altogether 93 polymorphisms were detected in the re-sequencing of these two regions, corresponding to one SNP approximately every 360 bp.

To be able to group genetically similar sires for the QTL for PY a 93 marker haplotype window (PY93) covering the region from 88.29 to 88.75 Mb was defined (Figure 1 and Figure 2). Haplotype classification within PY93 was performed based on phased chromosomes for the five re-sequenced sires (Table 2) and other NRC sires for which genotypes were available. The haplotypes identified in re-sequenced sires were denoted PY93_A, PY93_B, PY93_C, PY93_D and PY93_E, where PY93_A had the highest frequency and PY93_D the lowest. Each of these five haplotypes within the PY93 haplotype window was characterized by its unique set of alleles for the 93 markers. Both re-sequencing and haplotype classification indicated that 1606 Frasse was homozygous for the haplotype PY93_C, while his sons 1893 Rud and 2005 Smidesang both were heterozygous with one copy of this haplotype each. Among the 2601 sires for which genotypes and phenotypes were available, sires with at least one copy of the haplotype PY93_C, likely descendents from 1606 Frasse, had daughters with significantly elevated PY compared with remaining genotyped sires (two sample t-test gave p-value of 2.87E^-5 ^after correcting phenotypic values for relationship matrix and grandsire effect). The haplotype PY93_C was the only haplotype found in the re-sequenced sires that had a significant association with elevated PY (p-value < 0.01), motivating further characterization of this haplotype by use of re-sequencing data.

**Table 2:** Haplotype classification for the PY93 haplotype window

Animal	Haplotype 1 (PF)	PY (p-value)	Haplotype 2 (PF)	Effect (p-value)
1606 Frasse	PY93_C (0.137)	E (2.87^-5^)	PY93_C (0.137)	E (2.87^-5^)
1893 Rud	PY93_C (0.137)	E (2.87^-5^)	PY93_D (0.070)	-
2005 Smidesang	PY93_C (0.137)	E (2.87^-5^)	PY93_B (0.146)	R (5.35^-13^)
2636 Vik	PY93_B (0.146)	R (5.35^-13^)	PY93_E (0.025)	-
3454 J. Steinsvik	PY93_A (0.197)	-	PY93_E (0.025)	-

Haplotype 1 and haplotype 2 are given for NRC animals 1606 Frasse, 1893 Rud, 2005 Smidesang, 2636 Vik and 3454 J. Steinsvik for the PY93 haplotype window. Population frequencies (PF) were found based on phased chromosomes of 2601 NRC animals. Elevated (E) or reduced (R) protein yield (PY) is indicated when found significantly differing from the population mean (p-value < 0.01).

Comparison of the DNA sequence of the haplotype PY93_C with the DNA sequence of other haplotypes found in the re-sequenced sires revealed 6 polymorphisms in coding regions of casein genes *CSN2 *and *CSN1S2 *or in their 2000 bp 5'-flanking promoter regions (Table 3). Positive and negative alleles were assigned for these polymorphisms, with a positive allele defined as one found in PY93_C and therefore associated with increased PY (Table 3). The only non-synonymous substitution detected within the genes *CSN2 *and *CSN1S2 *was in amino acid 82 in *CSN2 *(Ref NM_181008.2), previously reported in NRC by Nilsen *et al*. [7]. This substitution has been reported to have differing effects on PY in various cattle breeds and is therefore not likely to be a significant causal polymorphism [8,38-40]. The re-sequencing also detected a silent substitution (C>T) in amino acid 125 of *CSN2 *(Ref NM_181008.2), previously reported by Lien *et al*. [5].

**Table 3:** Polymorphisms associated with protein yield

Btau_4.0 (bp)	Gene	Region	TPP *	NT [+/-]	TP [+/-]	PP (bp)
88331023	*CSN2*	Exon	125	[T/C] ^†^	P	
88331153	*CSN2*	Exon	82	[C/A] ^‡^	[P/H]	
88338754	*CSN2*	Promoter		[G/A]		-367
88339252	*CSN2*	Promoter		[T/A]		-865
88409015	*CSN1S2*	Promoter		[C/A]		-7
88410860	*CSN1S2*	UTR		[C/T]		

Btau_4.0 position [31], gene, description of region (promoter, exon or UTR), translated protein position (TPP), polymorphic nucleotide (NT) alleles denoted as positive or negative [+/-], polymorphic translated protein (TP) alleles denoted as positive or negative [+/-] and promoter position (PP) relative to transcription initiation site. * Translated protein position in Ref NM_181008.2 for CSN2 and Ref NM_ 174528.2 for CSN1S2. † Polymorphism previously described in Lien *et al*. [5]. ‡Polymorphism previously described in Nilsen *et al*. [7].

Of greater interest, a SNP (A>C) was detected in the promoter region of *CSN1S2 *at -7 bp relative to the transcription initiation site. The SNP, which has previously been reported by Schild and Geldermann [14], was positioned three base-pairs downstream of a CCAAT motif stretching from -14 to -10 bp. In the mammary gland the transcription factor C/EBP β functions as an enhancer of transcription by binding to CCAAT motifs and is crucial for transcription of casein genes [41-43]. It is possible that the polymorphism described here affects the binding affinity of enhancers to the CCAAT motif, and thereby affect transcription efficiency of *CSN1S2*.

**Figure 3:**
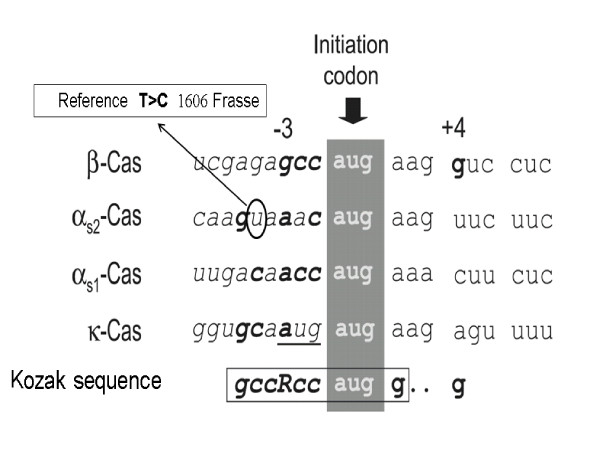
**A novel polymorphism in the Kozak sequence of α_S2_-casein**. A novel single-nucleotide polymorphism was discovered in a motif known to regulate efficiency of translation (the Kozak sequence) [45] in the 5'-flanking UTR of CSN1S2 coding for α_S2_-casein. The polymorphic nucleotide is indicated and alleles found in the reference sequence and in the Holstein-Fresian Bull 1606 Frasse are presented. The figure is a modification of the figure presented in Bevilacqua *et al*. [44].

A second SNP (T>C), that has not previously been reported, was detected in the 5'- UTR of the gene *CSN1S2 *at position -5 bp distant from the initiation codon in the sequence GYAAACatgG (Figure 3), and could directly influence translation of α_S2_-casein coded for by *CSN1S2*. Bevilacqua *et al*. [44] found that while transcripts from all four casein genes are found at similar concentrations in mammary tissue, translated α_S2_-casein and κ-casein are found in much lower concentrations in cow milk than α_S1_-casein and β-casein. The 5'- UTR sequence for the four caseins were strictly conserved between cattle, sheep and goat, and they suggested that variation in the Kozak consensus sequence (GCCRCCatgG [45]) might be the cause of the observed variation in translational efficiency between casein genes (Figure 3). Matching well with the higher protein levels associated with the PY93_C haplotype from 1606 Frasse; the C allele found in PY93_C was in better accordance with the Kozak consensus sequence than the alternative T allele, and therefore expected to produce a more efficient translation initiation site within the *CSN1S2 *transcript. Work is in progress to deduce functionality of the detected polymorphisms on the transcription and translation of *CSN1S2 *by expression profiling and quantitative determination of α_S2_-casein in milk.

### Long range haplotypes affecting clinical mastitis

Highest test scores for CM1, CM2 and CM3 from both single-marker and haplotype association mapping were found within the genomic interval 89 to 91 Mb on BTA6 (Table 1). To be able to group genetically similar sires for the QTL for CM a 48 marker haplotype window (CM48) covering the genomic interval 89.4 to 90.6 Mb was defined (Figure 1 and Figure 2). This interval contained the SNPs producing the highest test scores from association mapping for CM. Haplotype classification within CM48 was performed based on phased chromosomes for NRC sires for which genotypes were available. The sire 1606 Frasse was homozygous for the CM48 haplotype window, and sires with at least one copy of the haplotype found in 1606 Frasse had daughters with significantly elevated mastitis susceptibility compared with remaining genotyped sires (two sample t-test gave p-value of 2.03E^-5 ^after correcting phenotypic values for relationship matrix and grandsire effect).

As can be seen from Figure 1 and Figure 2, the 1606 Frasse haplotype was not markedly disrupted by recombination between the haplotype windows PY93 and CM48. This means that although there were no high LD between SNPs showing high association with PY and SNPs showing high association with CM in the genotyped population as a whole, there were strong correlations between these SNPs within individuals holding a copy of the haplotype from Frasse 1606 for this region. As previously noted the haplotype from Frasse 1606 was also significantly associated with higher levels of protein content in milk [5,7], which might partly explain the observed genetic correlation between PY and CM in NRC [1].

In contrast to the QTL for PY which gave very high test scores for potent candidate genes (Figure 1), results for CM were much more dispersed (Figure 2). SNPs strongly associated with CM were not concentrated to a few specific genes, meaning that a number of genes in the QTL region for CM could harbor polymorphisms potentially affecting mastitis susceptibility. Previously the genes *MUC7 *and *IGJ *have been proposed as candidate genes for mastitis susceptibility in this region [6], but elevated test scores for CM were not found in or around these two genes in the current study (Figure 2).

The highest test scores for CM were found within and surrounding the three genes *SLC4A4*, *GC *and *ADAMTS3*. The first of these (*SLC4A4*) codes for a sodium bicarbonate co-transporter involved in maintaining normal blood pH [46,47], the second (*GC*) encodes the main carrier protein of vitamin D in plasma, and finally the third gene (*ADAMTS3*) shows high similarity with *ADAMTS2*, which codes for a pro-collagen N-proteinase [48]. A cluster of genes coding for the CXC chemokines *IL8*, *CXCL5*, *PF4 *and *CXCL2 *are positioned around 92 Mb, quite close to the highest scoring region for CM. CXC chemokines are important pro-inflammatory mediators and might therefore contain variation affecting mastitis susceptibility. Expression levels of some of these CXC chemokines have been shown to increase in bovine mammary gland cell lines after exposure to bacterial cell wall components [49-52], underlining their relevance as candidate genes in this QTL region.

## Conclusion

Highest test scores from association mapping for PY were found within and surrounding the casein genes *CSN2 *and *CSN1S2*. Haplotype classification and high-coverage re-sequencing data indicated that the Holstein-Friesian bull 1606 Frasse, imported into the NRC cattle population in the 1970s, was homozygous for a haplotype encompassing these two genes. As previously suggested the haplotype from 1606 Frasse was significantly associated with elevated PY and selection for milk production traits is likely to have increased the frequency of this haplotype in the NRC population [5]. Data available from high throughput re-sequencing allowed for molecular characterization of the haplotype from 1606 Frasse, and plausible causal polymorphisms were detected in a regulatory element in the promoter region of the gene *CSN1S2 *as well as in a motif that regulates translation efficiency of *CSN1S2 *[44,45].

It was further shown that the long range haplotype from 1606 Frasse is highly conserved in the NRC population for the region spanning the two QTLs affecting PY and CM on BTA6. The positive effect on milk protein content and the negative effect on mastitis susceptibility of this haplotype might partly explain the observed genetic correlation between these two traits in NRC [1]. Our results emphasize the importance of inclusion of mammary gland health in dairy cattle breeding goals.

## Methods

### Animals and phenotypes

NRC is an admixed breed formed from Norwegian breeds and imported animals from other Nordic countries. Norway has a dairy herd recording system which has included veterinary reported clinical mastitis (VRCM) since 1975 [53]. Records of VRCM in the periparturient period (-15 to 30 days post partum) of first (CM1), second (CM2) and third (CM3) lactation were available from GENO Breeding and AI Association [54] as daughter-yield-deviations (DYDs) for NRC sires. DYDs are phenotypic values corrected for non-genetic effects believed to affect the trait, and a sire with a high DYD value for CM has daughters with increased susceptibility to CM. Here records of VRCM were retrieved as a binary trait for daughters of sires from paternal half-sib families, yielding a large number of records per sire and a reduction in variance due to environmental effects compared with other designs [55]. The mastitis traits CM1, CM2 and CM3 are described in Table 4 together with heritabilities and genetic correlations reported by Svendsen and Heringstad [54].

**Table 4:** The mastitis traits

	Number of records		Heritability and genetic correlation		
Trait	Daughters	Sires	CM1	CM2	CM3
CM1	1,755,649	2,596	0.03	0.74	0.68
CM2	1,256,887	2,532	-	0.02	0.85
CM3	805,376	2,440	-	-	0.02

Trait and number of sire and daughter records for the clinical mastitis traits CM1, CM2 and CM3 are presented. The final columns give heritabilities (on the diagonal) and genetic correlations reported by Svendsen and Heringstad [54].

Records of PY were also available from GENO Breeding and AI Association as DYDs for NRC sires and were retrieved as 3,481,538 daughter records of 2,596 sires from paternal half-sib families. A sire with a high DYD value for PY has daughters with increased PY. For both traits (CM and PY) number of daughter records per sire was highly variable and influenced by a small number of elite sires with a large number of daughters. DNA samples for sires included in this study were extracted from semen available from GENO Breeding and AI Association.

### Re-sequencing of candidate region and SNP detection

Sequence capture using a Roche-Nimblegen product was performed to isolate the region of interest on BTA6. Roche NimbleGen designed and manufactured a 5 Mb sequence capture array targeting BTA6 coordinates 88-97 Mb (385 K NimbleGen sequence capture arrays), standard repeat masking (approximately 50% of the sequence was masked) was applied in the design with 80% of the targeted bases being within a 100 bp window of the final probe set. Sequence capture library construction was performed on seven samples, four NRC sires (2005 Smidesang, 10243 Rishaugen, 10263 Frestad and 10553 Nordbø) and three pools of old Norwegian breeds. Samples were sequenced using a 454 GS-FLX platform with the number of reads generated from each sample ranging from 97-460 k. Sequence data from all samples was aligned to the BTA_4.0 reference genome [31] using the MOSAIK software package and default alignment parameters [32], yielding an average coverage of 5× for the region of interest.

SNP detection was performed with GigaBayes [33]. Criteria for filtering SNPs included minimum number of reads of each variant in non-coding regions (≥2), minimum number of reads of the variant differing from the reference sequence for coding region (≥2), GigaBayes score (≥0.95) and minimum distance to closest SNP (>5 bp). SNPs positioned in homopolymer regions (>5 bp) were also rejected. After filtering 269 SNPs remained.

### Genotype datasets

Four genotype datasets were combined to yield a final dataset of 556 SNPs. The 269 SNPs identified by re-sequencing of the candidate region by Roche NimbleGen sequence capture and 454 GS-FLX sequencing were genotyped in 768 sires from paternal half-sib families using the Sequenom MassARRAY system (dataset 1). Genotypes were also retrieved for 84 SNPs for 2589 NRC sires from paternal half-sib families genotyped with the Affymetrix 25 k MIP array (dataset 2) and for 198 SNPs for 1092 NRC sires from paternal half-sib families genotyped with the Illumina Bovine SNP50 BeadChip (dataset 3). Finally, genotypes for 102 SNPs in the genomic region 86-90.4 Mb on BTA6 were retrieved for 1133 sires [6] (dataset 4). Minimum and maximum number of SNPs a sire could be genotyped for were 84 and 556, respectively. Some SNPs were present in more than one dataset (table 5).

**Table 5:** Genotype datasets

Dataset	Source	SNPs	Unique SNPs	Sires
1	454 re-sequencing	269	269	768
2	25 k	84	35	2589
3	50 k	198	121	1092
4	Nilsen *et al*., 2009 [7]	103	96	1133

The four genotype datasets that contributed to the final genotype dataset of 556 SNPs used for association mapping are summarized. For each dataset source of SNPs, number of SNPs used, number of SNPs unique for the dataset and number of sires genotyped for the SNPs are given.

### Data correction and imputation

The genotyped data were checked for mendelian errors and based on the observed results, a cut-off of 4% was set to identify samples not fitting the pedigree. Pedigree errors were resolved by either identifying a new sire or setting parental information to unknown for the affected animal. New sires were assigned when the number of mendelian errors was equal to or lower than the background (0.4%) and there was only one candidate.

Linkage analysis, haplotyping and imputation were conducted with CRIMAP [56] and locally developed software to combine the four datasets and fill in untyped genotypes. The phasing procedure was implemented based on the six rules algorithm presented by Qian *et al*. [57], with modifications to fit half-sib families with missing data. Imputation in paternal haplotypes was performed by assuming no recombination between informative markers of the same phase. Imputation in haplotypes where no parental genotype information was available was performed by searching the rest of the dataset for equal haplotypes at surrounding informative positions and imputing when the untyped genotype could be decided uniquely. The final dataset contained genotypes for 556 SNPs in the BTA6 86-97 Mb genomic region for 2601 sires, with on average 1.2% of SNPs missing for each individual sire.

### Single-marker association mapping

Single-marker association mapping for CM1, CM2, CM3 and PY were performed for all SNPs. The mixed model was:

Here phenotypic value P is DYD of sire i weighted by number of daughters, g is fixed effect of grandsire j, a is random effect of sire i where co-variance structure between sires is determined from pedigree relationships, m is random effect of genetic marker k and e is an error term. Estimation was conducted with the ASREML software [58].

### Haplotype association mapping

Pair-wise LD measure r^2 ^was found for all SNP pairs with the Haploview 4.1 software [59] and haplotype blocks were defined by the method described in Gabriel *et al*. [36] (GAB) and by the four gamete rule [37](GAM). A perl script was written to classify sires according to haplotypes for each of the defined haplotype blocks. The classification into GAB and GAM blocks were implemented in haplotype association mapping for CM1, CM2, CM3 and PY. The mixed model was:

Here phenotypic value P is DYD of sire i weighted by number of daughters, g is fixed effect of grandsire j, a is random effect of sire i where co-variance structure between sires is determined from pedigree relationships, h is random effect of haplotype k and e is an error term. Estimation was conducted with the ASREML software [58].

### Test score and multiple testing

LRT scores were calculated as two times the log-likelihood (LogL) ratio. LogL ratios were obtained with the ASREML software [58] for each SNP or haplotype as the difference between the LogL of a model containing the effect of the SNP or haplotype and the LogL of a model not containing this effect. LRT scores were expected to be distributed as a mixture of two χ^2 ^distributions with 0 and 1 degree of freedom. To correct for multiple testing the significance threshold was corrected with the effective number of independent tests (Meff_G_) [60]. Meff_G _was found to be 208 for the 556 genotyped SNPs, corresponding to an adjusted p-value of 4.8E^-5 ^(0.01/208 = 4.8E^-5^) and a LRT score of 15.42.

MacLeod *et al*. [61] demonstrated that including effect of sire based on pedigree relationships reduces the number of false positives in association studies.

### Genome re-sequencing and detection of polymorphisms

Genome re-sequencing of five NRC sires (1606 Frasse, 2636 Vik, 3454 J. Steinsvik, 2005 Smidesang and 1893 Rud) was performed on an Illumina GAIIx platform in order to characterize the haplotypes of these animals and identify putative causal polymorphims. Reads were generated as 2 × 108 paired-ends, coverage was 10 Gb for 1606 Frasse and 2636 Vik, and 4 Gb for 3454 J. Steinsvik, 2005 Smidesang and 1893 Rud. The FASTQ/A Clipper program from the FASTX-Toolkit [62] was used to remove adapter sequence and to discard reads based on average quality score (<10) or untyped bases (>7 Ns). Sequence data was assembled by mapping reads to the BTA_4.0 reference genome [31] using the BWA software package [63] and dafault alignment parameters. Polymorphism detection was performed with SAMtools [64]. Criteria for filtering included minimum number of reads (≥2), maximum number of reads (≤100), minimum number of reads of the variant differing from the reference sequence (≥2) and minimum RMS mapping value (≥25). Polymorphisms positioned in homopolymer or repeat regions were discharged.

## Authors' contributions

MS bioinformatics on 454 sequence data, statistical analyses and writing the manuscript. HG joining datasets by haplotyping and imputation. MK conducted and coordinated molecular genetics work. ST bioinformatics on Illumina sequence data. MS provided DYDs and pedigree information. BJH assisted in drafting the manuscript. SL conceived of and coordinated the study and assisted in drafting the manuscript. All authors helped finalize the manuscript and read and approved of the final version. The authors declare that they have no competing interests.

## Supplementary Material

Additional file 1 **Genotyped single-nucleotide polymorphisms**. The 556 single nucleotide polymorphisms (SNPs) genotyped in this study are presented by position, alleles and missing genotype percentage. Click here for file

Additional file 2 **Linkage disequilibrium**. Pairwise linkage disequilibrium (LD) between all pairs of the 556 single nucleotide polymorphisms (SNPs) genotyped in this study presented by *Bos taurus *chromosome 6 (BTA6) positions (complete LD is indicated in white). Click here for file
