# Supplementary material for: Molecular characterization of a long range haplotype affecting protein yield and mastitis susceptibility in Norwegian Red cattle
Source: BMC Genet. 2011 Aug 11;12:70. doi: 10.1186/1471-2156-12-70 (PMC3171720; doi:10.1186/1471-2156-12-70)
Supplement: Additional file 1 — Genotyped single-nucleotide polymorphisms. The 556 single nucleotide polymorphisms (SNPs) genotyped in this study are presented by position, alleles and missing genotype percentage. [file 1471-2156-12-70-S1.PDF]

Additional file 1

Table A1 – Genotyped single-nucleotide polymorphisms

The 556 single nucleotide polymorphisms (SNPs) genotyped in this study are presented by position, alleles and missing genotype percentage.

SNP	Position (bp)	A1	A2	Missing%	SNP	Position (bp)	A1	A2	Missing%
BTA-76946	86,091,437	A	G	0.0069	BTA-77101	88,268,695	A	G	0.0062
ss86309338	86,091,957	A	C	0.0062	ss86217839	88,291,433	A	G	0.1207
rs43703008	86,104,461	C	G	0.0065	ss86217840	88,291,473	C	G	0.0042
rs41654417	86,123,130	C	T	0.0042	ss86217841	88,295,268	C	T	0.0081
rs41654416	86,127,539	G	T	0.0065	ss117968347	88,302,639	A	G	0.0081
rs29001782	86,128,027	A	G	0.0069	ss290490306	88,305,616	A	G	0.0050
ss61506487	86,365,126	A	C	0.0065	ss86217842	88,306,150	C	G	0.0073
ss61557722	86,434,938	A	G	0.0088	rs43703010	88,307,280	A	G	0.1200
ss61491570	86,467,724	A	G	0.0115	ss86217843	88,307,439	A	G	0.0042
rs41618641	86,613,445	A	G	0.0058	BTA-115153	88,315,660	A	G	0.0054
ss86291015	86,671,685	A	G	0.0108	ss117968764	88,326,006	A	G	0.0119
ss86297176	86,712,348	A	G	0.0119	ss86217844	88,330,008	C	T	0.0119
rs29010229	86,810,566	A	T	0.0119	ss86217845	88,330,265	G	T	0.0115
rs41570706	86,908,337	A	T	0.0100	rs43703013	88,330,987	C	G	0.0027
BTA-113299	86,965,533	C	G	0.0062	ss86217846	88,331,026	C	T	0.0054
ss117967957	87,103,072	A	C	0.0138	rs43703011	88,331,153	A	C	0.0111
rs29011726	87,242,360	C	T	0.0012	ss86217847	88,332,840	G	T	0.0111
rs29011727	87,242,379	A	G	0.0012	ss86217848	88,333,146	A	G	0.0119
rs29011728	87,242,429	A	G	0.0012	ss86217849	88,333,706	C	T	0.1027
ss86307579	87,255,540	A	G	0.0012	ss86217850	88,335,937	A	G	0.0119
ss86291546	87,370,506	A	G	0.0104	ss86217851	88,337,212	A	G	0.0023
ss61466227	87,480,010	A	C	0.0150	ss86217852	88,337,966	A	G	0.0023
ss86324844	87,663,732	A	C	0.0096	ss86217853	88,338,919	A	T	0.0119
ss86317213	87,879,379	A	G	0.0058	ss86217854	88,339,983	A	T	0.0023
rs41610994	87,903,788	C	T	0.0223	ss86217855	88,340,058	C	T	0.0058
rs41610993	87,903,902	C	T	0.0050	ss117968472	88,350,096	A	G	0.0058
ss61516066	87,904,282	A	G	0.0038	ss117968030	88,370,146	A	C	0.0054
rs29010267	87,989,290	A	G	0.0038	ss86217856	88,377,887	A	G	0.0104
BTA-77094	87,989,565	A	G	0.0038	ss86217857	88,378,201	C	T	0.0104
ss290490277	88,140,290	A	G	0.0100	ss86217858	88,378,904	C	T	0.0104
rs29015040	88,160,178	A	T	0.0012	ss117968093	88,391,613	A	C	0.0050
rs29015039	88,160,213	C	G	0.0012	ss86217859	88,407,310	A	G	0.0050
ss290490293	88,233,640	A	C	0.0185	ss86217860	88,407,939	C	T	0.0119
ss117968170	88,263,656	A	G	0.0100	ss86217861	88,408,758	A	G	0.0042

SNP	Position (bp)	A1	A2	Missing%	SNP	Position (bp)	A1	A2	Missing%
ss86217862	88,410,501	A	G	0.0104	ss86217887	88,532,740	A	G	0.0135
ss86217863	88,412,404	C	T	0.0108	ss86217888	88,532,923	C	T	0.0031
ss86217864	88,413,712	C	T	0.0104	ss86217889	88,532,930	A	C	0.0031
ss86217865	88,415,611	A	G	0.0108	ss86217890	88,533,205	G	T	0.0031
ss86217866	88,415,827	A	G	0.0023	ss86217891	88,533,423	A	G	0.0031
ss86217867	88,416,651	A	G	0.0042	ss86217892	88,533,570	A	G	0.0127
ss86217868	88,419,759	A	T	0.0038	ss86217893	88,533,625	A	G	0.0031
ss86217869	88,422,590	C	T	0.0042	ss86217894	88,534,065	C	G	0.0031
ss86217870	88,423,433	C	T	0.0111	rs29024681	88,537,898	A	G	0.0031
ss86217871	88,426,655	A	G	0.0104	rs29024683	88,537,969	A	G	0.0027
ss86217872	88,427,363	C	T	0.0104	rs29024684	88,538,027	A	C	0.0031
ss86217873	88,427,486	A	G	0.0104	rs29024685	88,538,077	A	G	0.0031
ss117968525	88,427,761	A	G	0.0142	ss290490349	88,544,997	G	A	0.0027
rs41588955	88,470,657	A	G	0.0108	ss290490350	88,545,414	A	G	0.0027
rs41588953	88,470,917	C	T	0.0073	ss290490351	88,545,614	A	G	0.0150
ss117968780	88,473,588	A	G	0.0046	ss290490361	88,579,785	C	G	0.0027
ss290490497	88,500,334	C	A	0.0046	rs29025858	88,657,039	A	T	0.0111
ss86217874	88,505,291	A	G	0.0181	ss290490422	88,723,114	A	G	0.0085
ss86217875	88,505,604	C	T	0.0327	ss61465597	88,724,564	A	C	0.0119
rs43703014	88,505,736	A	T	0.0050	ss290490426	88,731,394	A	G	0.0088
rs41588950	88,508,849	A	G	0.0319	ss290490427	88,731,444	G	A	0.0154
rs41588946	88,508,981	G	T	0.0123	ss86217895	88,757,210	C	T	0.0300
rs41588945	88,509,069	C	T	0.0323	ss86217896	88,761,333	C	T	0.0150
rs41588944	88,509,123	C	T	0.0323	ss99307233	88,761,588	C	G	0.0150
ss290490338	88,512,065	G	A	0.0323	ss86217897	88,761,753	A	C	0.0115
ss86217876	88,519,758	A	T	0.0161	ss86217898	88,761,866	A	G	0.0058
ss86217877	88,520,726	A	G	0.0031	ss86217899	88,761,904	A	T	0.0069
ss86217878	88,520,893	G	T	0.0323	ss86217900	88,762,614	C	T	0.0058
ss86217879	88,520,981	C	T	0.0319	ss99307234	88,762,822	A	T	0.0119
ss86217880	88,521,023	C	T	0.0319	ss290490538	88,784,759	A	G	0.0196
ss86217881	88,528,999	A	C	0.0319	ss86312906	88,806,897	A	C	0.0277
ss86217882	88,530,213	C	T	0.0323	ss290490478	88,874,119	G	A	0.0115
ss86217883	88,531,652	A	G	0.0054	ss86217901	88,905,399	C	T	0.0127
ss290490513	88,532,297	G	A	0.0127	ss86217902	88,905,683	A	G	0.0115
rs43703015	88,532,298	C	T	0.0031	ss86217903	88,905,911	A	G	0.0154
rs43703016	88,532,334	A	C	0.0031	ss86217904	88,907,156	C	G	0.0204
rs43703017	88,532,354	A	G	0.0031	ss290490492	88,907,992	C	G	0.0115
ss86217884	88,532,395	A	G	0.0081	rs29019575	88,946,761	A	G	0.0146
ss86217885	88,532,403	A	T	0.0031	ss117968738	88,983,535	A	G	0.0019
ss86217886	88,532,715	C	T	0.0031	rs41655346	88,987,517	A	T	0.0073

SNP	Position (bp)	A1	A2	Missing%	SNP	Position (bp)	A1	A2	Missing%
rs41655347	88,990,898	A	C	0.0073	ss290490289	90,139,654	A	G	0.0115
ss290490271	89,030,092	G	A	0.0054	ss290490290	90,172,727	G	A	0.0085
ss86326721	89,030,229	A	G	0.0100	ss61489238	90,184,757	A	G	0.0073
rs29010354	89,081,281	A	G	0.0100	rs29025895	90,241,567	A	G	0.0081
ss290490503	89,090,122	C	A	0.0081	ss86302265	90,259,358	A	G	0.0138
ss290490272	89,135,182	A	G	0.0050	rs41629222	90,284,840	A	T	0.0100
ss290490273	89,135,353	G	A	0.0058	BTA-122716	90,284,888	A	G	0.0088
ss61557767	89,150,759	A	G	0.0131	ss61528083	90,288,991	A	C	0.0058
ss290490274	89,195,672	C	G	0.0027	ss290490291	90,312,723	G	A	0.0062
ss86341106	89,212,072	A	G	0.0135	ss61562683	90,317,539	A	C	0.0077
rs29010419	89,274,692	A	G	0.0092	ss290490292	90,327,562	A	G	0.0054
ss290490275	89,339,428	C	A	0.0104	ss61524397	90,356,013	A	G	0.0108
ss86285294	89,355,142	A	G	0.0111	ss290490294	90,374,858	A	G	0.0054
rs41588980	89,355,672	A	G	0.0115	ss290490295	90,380,155	G	A	0.0100
rs41655357	89,369,237	A	C	0.0054	ss86297489	90,415,520	A	G	0.0096
rs41655356	89,369,291	C	T	0.0119	ss290490493	90,466,078	A	G	0.0081
ss290490276	89,374,004	G	A	0.0150	ss86317874	90,485,680	A	G	0.0085
ss290490504	89,402,237	G	A	0.0131	rs41629221	90,508,812	A	C	0.0085
ss290490278	89,403,467	A	C	0.0069	ss290490296	90,516,475	A	T	0.0073
ss290490279	89,428,900	A	C	0.0165	ss290490297	90,518,605	G	A	0.0085
ss290490280	89,482,383	C	G	0.0065	ss61507506	90,564,544	A	G	0.0073
rs43474199	89,510,017	A	C	0.0088	ss290490298	90,600,152	A	G	0.0038
rs29022799	89,603,520	A	G	0.0073	ss290490299	90,600,253	G	A	0.0073
ss61524338	89,623,896	A	C	0.0012	ss290490300	90,605,540	A	G	0.0096
ss290490281	89,625,793	A	G	0.0108	ss290490301	90,608,741	C	G	0.0031
ss290490282	89,650,093	A	T	0.0100	rs29024027	90,642,598	C	G	0.0096
ss290490283	89,650,183	G	A	0.0104	rs29024026	90,644,772	A	C	0.0465
ss86278591	89,668,440	C	G	0.0081	ss61522200	90,670,190	A	G	0.0035
ss290490284	89,702,619	C	G	0.0081	rs43052931	90,715,456	A	G	0.0058
ss38332444	89,730,160	A	G	0.0123	ss290490302	90,724,105	A	G	0.0065
ss86337596	89,774,922	A	G	0.0104	ss290490303	90,725,368	A	G	0.0065
ss290490528	89,808,406	G	A	0.0088	rs43052940	90,737,717	A	G	0.0100
rs43338539	89,838,827	A	G	0.0092	ss290490304	90,919,075	C	G	0.0127
ss290490285	89,921,855	A	G	0.0096	ss290490305	90,919,904	C	G	0.0027
rs43338568	89,926,345	A	G	0.0100	rs42932743	90,990,506	A	C	0.0100
ss290490286	89,942,988	G	A	0.0077	ss61557794	91,047,325	A	C	0.0088
ss86296213	90,008,099	A	G	0.0100	ss290490307	91,064,920	G	A	0.0058
ss290490287	90,011,530	A	G	0.0111	ss290490529	91,074,321	G	A	0.0085
ss290490288	90,012,190	A	G	0.0108	ss290490308	91,089,897	G	A	0.0085
rs42766480	90,075,263	A	G	0.0058	ss290490309	91,106,225	G	A	0.0104

SNP	Position (bp)	A1	A2	Missing%
ss290490530	91,121,513	A	G	0.0088
ss290490494	91,121,785	A	G	0.0146
BTA-77209	91,138,928	A	T	0.0104
ss61523674	91,140,233	A	G	0.0092
ss290490505	91,160,463	A	G	0.0081
rs41869408	91,190,937	A	G	0.0108
ss290490310	91,199,714	G	A	0.0108
rs41870471	91,303,460	A	C	0.0088
ss290490311	91,365,786	A	G	0.0081
ss290490312	91,387,149	A	C	0.0100
rs42149268	91,417,374	A	G	0.0081
ss290490506	91,472,826	G	A	0.0100
ss290490507	91,493,203	A	C	0.0085
ss61550746	91,553,825	A	G	0.0127
ss290490313	91,571,393	C	G	0.0135
ss290490314	91,599,731	C	A	0.0135
ss61557678	91,692,660	A	G	0.0135
rs29012368	91,747,096	A	G	0.0127
ss290490315	91,815,688	G	A	0.0031
ss290490495	91,831,341	A	C	0.0104
rs43471504	91,874,621	A	G	0.0150
rs43471476	91,897,799	A	C	0.0050
ss86334878	91,918,867	A	G	0.0092
ss290490316	91,933,203	G	A	0.0100
ss61557674	91,961,905	A	G	0.0085
ss290490317	91,966,668	A	C	0.0062
ss86311372	92,030,874	A	G	0.0062
ss290490318	92,060,584	A	C	0.0100
ss290490508	92,060,620	G	A	0.0119
ss290490319	92,060,723	A	G	0.0108
ss290490320	92,078,268	G	A	0.0108
BTA-76997	92,095,682	A	G	0.0100
ss61557664	92,150,444	A	G	0.0092
ss290490321	92,157,186	A	G	0.0042
ss86339292	92,179,319	C	G	0.0046
ss290490322	92,181,027	A	G	0.0100
ss86339125	92,216,838	A	G	0.0077
ss290490323	92,219,093	C	G	0.0085
rs29016177	92,240,040	A	G	0.0092
rs42959067	92,274,348	A	G	0.0046
ss61557797	92,307,060	A	G	0.0081
rs42581544	92,434,963	A	G	0.0111
ss290490496	92,445,535	C	A	0.0131
ss61496193	92,473,530	A	C	0.0085
ss86285114	92,517,198	A	G	0.0023
ss290490324	92,564,474	A	C	0.0104
ss290490325	92,564,611	G	A	0.0123
ss86300582	92,646,173	A	G	0.0100
ss290490326	92,648,026	A	C	0.0111
ss290490327	92,767,066	A	G	0.0111
ss290490328	92,767,192	G	A	0.0135
ss86335839	92,767,317	A	G	0.0135
ss290490329	92,769,567	A	G	0.0131
ss290490330	92,784,117	G	A	0.0135
ss290490331	92,784,162	A	G	0.0115
ss290490509	92,786,144	A	G	0.0119
ss290490510	92,786,324	A	C	0.0119
ss290490511	92,787,256	A	C	0.0138
ss61568524	92,788,188	A	G	0.0246
ss290490512	92,791,899	G	A	0.0119
ss290490332	92,827,164	G	A	0.0127
ss86310987	92,835,501	A	C	0.0115
rs42773532	92,854,146	A	G	0.0123
ss86333471	92,886,879	A	G	0.0135
ss290490333	92,895,286	A	G	0.0115
rs42775634	92,914,806	A	G	0.0096
ss61496362	92,949,328	A	G	0.0115
rs42592169	93,002,336	A	G	0.0050
ss290490334	93,030,857	A	G	0.0115
rs42592200	93,031,311	A	G	0.0111
ss86313553	93,055,656	A	G	0.0135
ss290490335	93,059,912	A	G	0.0127
ss290490336	93,061,634	A	G	0.0127
ss290490337	93,102,712	G	A	0.0142
BTA-95635	93,124,191	A	G	0.0127
ss290490339	93,124,587	A	C	0.0092
ss290490340	93,124,691	G	A	0.0315
rs42582518	93,155,307	A	G	0.0085
ss290490341	93,273,006	A	G	0.0088
ss290490342	93,283,723	A	G	0.0054

SNP	Position (bp)	A1	A2	Missing%	SNP	Position (bp)	A1	A2	Missing%
ss290490343	93,283,784	A	G	0.0108	ss290490517	94,064,479	A	C	0.0050
ss290490344	93,295,667	A	G	0.0519	ss290490499	94,065,214	A	G	0.0046
ss290490345	93,297,134	C	G	0.0135	ss290490518	94,081,399	A	T	0.0108
ss61493860	93,305,750	A	G	0.0077	ss117968835	94,082,193	A	C	0.0108
ss61569057	93,371,538	A	G	0.0138	ss46526537	94,129,168	A	G	0.0058
ss290490346	93,396,499	C	G	0.0023	rs41256838	94,129,426	A	G	0.0069
ss290490347	93,407,278	C	A	0.0119	ss290490369	94,203,179	A	G	0.0069
ss290490348	93,409,101	A	G	0.0119	ss86289048	94,204,423	A	G	0.0146
ss290490352	93,458,165	A	G	0.0119	ss290490532	94,220,618	G	A	0.0158
rs42615162	93,463,204	A	G	0.0119	ss290490519	94,223,260	A	G	0.0065
ss290490353	93,468,271	C	A	0.0185	ss290490520	94,223,635	A	G	0.0154
ss290490354	93,468,295	G	A	0.0119	ss290490370	94,251,020	G	A	0.0081
ss290490355	93,486,836	G	A	0.0123	ss290490521	94,259,201	A	G	0.0073
rs43479253	93,545,537	A	G	0.0131	ss46526588	94,267,998	A	G	0.0165
ss290490356	93,546,692	C	G	0.0096	ss290490522	94,272,032	A	G	0.0096
ss290490498	93,546,941	G	A	0.0108	ss290490523	94,272,044	A	G	0.0096
ss290490357	93,566,788	C	A	0.0108	ss290490524	94,286,726	A	G	0.0211
ss290490358	93,613,019	G	A	0.0031	ss86294120	94,293,113	A	C	0.0100
ss86316121	93,640,925	A	G	0.0054	ss86336873	94,318,084	A	G	0.0092
ss290490359	93,645,877	C	G	0.0196	ss290490371	94,336,752	G	A	0.0088
rs42553777	93,682,966	A	G	0.0096	ss290490372	94,336,772	A	G	0.0085
rs42553790	93,704,721	A	G	0.0200	ss290490373	94,336,828	G	A	0.0085
ss61495618	93,729,871	A	G	0.0246	ss290490374	94,337,075	G	A	0.0085
rs42992679	93,767,254	A	G	0.0161	ss290490375	94,339,238	A	G	0.0085
ss290490360	93,789,318	A	G	0.0208	ss290490376	94,343,946	C	G	0.0231
rs42555873	93,850,919	A	C	0.0177	ss290490377	94,369,447	G	A	0.0158
ss290490514	93,864,750	A	G	0.0104	rs43477315	94,384,509	A	G	0.0154
ss290490362	93,877,815	A	G	0.0104	ss290490378	94,409,488	A	G	0.0100
ss290490363	93,890,378	C	G	0.0104	ss290490379	94,413,736	A	G	0.0092
rs42553820	93,918,271	A	C	0.0069	ss290490380	94,414,384	G	A	0.0096
ss290490364	93,955,044	A	G	0.0288	ss86313930	94,433,956	A	G	0.0054
ss290490531	93,955,540	A	G	0.0042	ss290490381	94,487,721	G	A	0.0081
ss86291415	93,962,055	A	G	0.0104	ss290490382	94,487,844	A	G	0.0046
ss290490365	93,977,073	A	G	0.0300	ss290490383	94,526,163	G	A	0.0046
ss290490366	93,984,576	G	A	0.0185	ss290490384	94,540,023	A	G	0.0050
ss290490367	94,023,891	G	A	0.0085	ss290490385	94,541,850	A	G	0.0146
ss290490368	94,023,958	A	G	0.0131	ss290490386	94,541,909	C	A	0.0054
ss290490515	94,027,168	C	A	0.0092	ss61557749	94,544,954	A	G	0.0092
ss290490516	94,027,231	A	G	0.0092	ss290490387	94,607,514	A	T	0.0085
ss117967932	94,050,759	A	G	0.0092	ss290490388	94,610,441	C	G	0.0065

SNP	Position (bp)	A1	A2	Missing%
ss290490389	94,610,634	G	A	0.0069
ss290490390	94,622,612	A	G	0.0065
ss290490391	94,624,188	C	G	0.0054
ss86322902	94,640,252	A	G	0.0131
ss290490392	94,667,541	G	A	0.0158
ss290490393	94,674,676	A	G	0.0135
ss290490394	94,688,056	A	G	0.0088
ss290490395	94,688,324	G	A	0.0096
rs43475613	94,709,427	A	G	0.0096
ss290490533	94,716,379	A	G	0.0092
rs29016391	94,720,776	A	G	0.0092
rs29016392	94,723,804	A	C	0.0092
ss86341394	94,736,057	A	G	0.0092
rs29016270	94,737,420	A	G	0.0088
ss290490396	94,738,925	G	A	0.0181
ss290490397	94,740,736	A	G	0.0085
ss290490398	94,786,769	G	A	0.0092
ss86339206	94,788,665	A	G	0.0242
ss290490534	94,790,634	A	G	0.0081
ss86293643	94,827,334	A	G	0.0085
ss290490399	94,833,805	G	A	0.0123
ss290490535	94,856,096	C	G	0.0150
ss290490400	94,861,220	A	G	0.0154
ss290490401	94,866,365	C	A	0.0127
ss290490402	94,871,888	A	G	0.0108
ss290490403	94,872,070	G	A	0.0108
ss290490404	94,872,209	C	G	0.0108
ss117963883	94,872,476	A	G	0.0108
ss290490405	94,873,310	A	G	0.0108
BTA-77150	94,876,519	A	G	0.0115
ss290490406	94,890,932	A	G	0.0042
ss290490407	94,895,845	G	A	0.0154
ss290490408	94,929,113	C	G	0.0138
ss290490409	94,934,119	A	G	0.0062
BTA-77152	94,957,486	A	G	0.0115
ss86332222	94,977,130	A	G	0.0038
ss290490410	95,018,465	G	A	0.0135
ss290490411	95,025,716	G	A	0.0119
ss290490412	95,027,742	G	A	0.0127
ss290490413	95,027,990	A	G	0.0131
ss290490414	95,030,075	A	G	0.0127
ss290490536	95,035,373	G	A	0.0127
ss61557762	95,042,615	A	C	0.0473
ss290490415	95,054,092	G	A	0.0138
ss290490416	95,069,325	C	G	0.0115
rs43480110	95,080,504	A	G	0.0131
ss290490500	95,081,622	C	A	0.0092
ss290490417	95,094,642	G	A	0.0131
ss290490525	95,095,881	G	A	0.0131
ss290490526	95,095,929	A	C	0.0131
ss86330106	95,103,436	A	G	0.0131
ss290490418	95,125,409	G	A	0.0135
rs43470932	95,137,925	A	G	0.0127
rs43470953	95,163,007	A	G	0.0058
ss290490419	95,183,015	G	A	0.0058
ss290490420	95,217,082	G	A	0.0115
ss290490421	95,217,309	G	A	0.0108
ss61472560	95,229,149	A	G	0.0108
ss290490423	95,240,127	C	A	0.0108
BTA-109071	95,256,811	A	G	0.0138
ss290490424	95,257,839	A	T	0.0062
ss86286430	95,269,007	A	C	0.0127
ss290490425	95,272,273	G	A	0.0127
ss290490537	95,286,837	A	G	0.0123
ss290490527	95,313,175	G	A	0.0123
ss290490428	95,315,550	G	A	0.0123
ss290490429	95,349,924	G	A	0.0123
ss290490430	95,356,138	C	A	0.0115
ss290490431	95,357,823	A	G	0.0131
ss290490432	95,361,612	C	G	0.0115
ss290490433	95,381,933	A	G	0.0131
ss290490434	95,409,611	G	A	0.0100
ss290490435	95,410,456	C	G	0.0215
ss290490436	95,418,018	A	C	0.0219
ss290490437	95,510,680	C	G	0.0138
ss86332567	95,528,037	A	C	0.0085
ss117968857	95,643,454	A	G	0.0115
ss290490438	95,696,847	C	G	0.0135
ss86306932	95,704,460	A	G	0.0081
ss290490439	95,707,881	A	G	0.0135

SNP	Position (bp)	A1	A2	Missing%	SNP	Position (bp)	A1	A2	Missing%
rs29020798	95,739,958	A	C	0.0115	ss290490457	96,397,663	A	T	0.0108
rs29020799	95,740,171	A	T	0.0123	ss290490458	96,400,023	G	A	0.0085
rs29020800	95,740,392	A	C	0.0315	ss290490459	96,405,322	A	G	0.0027
rs43479594	95,770,022	A	G	0.0123	ss290490460	96,416,402	A	G	0.0131
ss290490440	95,779,152	G	A	0.0119	ss290490461	96,422,848	G	A	0.0038
rs43475934	95,800,600	A	G	0.0131	ss290490462	96,423,037	A	G	0.0365
ss61523677	95,840,994	A	G	0.0115	ss290490463	96,426,125	G	A	0.0100
ss61557804	95,925,105	A	G	0.0035	ss290490464	96,439,911	C	A	0.0100
ss61557805	95,958,447	A	G	0.0088	ss290490465	96,445,514	C	A	0.0115
rs42800221	95,984,333	A	C	0.0096	rs29014369	96,447,880	C	G	0.0165
rs42801113	96,023,301	A	G	0.0096	ss290490466	96,461,261	A	G	0.0065
ss86341675	96,063,579	A	G	0.0096	ss290490467	96,469,079	A	G	0.0077
ss290490441	96,068,950	A	G	0.0038	ss290490468	96,469,211	A	G	0.0169
ss290490501	96,086,729	C	G	0.0092	ss290490469	96,470,042	C	A	0.0131
ss290490442	96,120,611	G	A	0.0042	ss290490470	96,485,952	C	A	0.0131
BTA-77356	96,189,520	A	C	0.0123	ss290490471	96,511,422	G	A	0.0711
ss86289414	96,193,687	A	G	0.0119	rs43482362	96,513,910	A	G	0.0058
ss290490443	96,195,493	A	G	0.0092	ss290490472	96,534,488	C	G	0.0062
ss290490444	96,217,099	A	G	0.0096	ss290490473	96,590,026	G	A	0.0088
BTA-77352	96,217,245	A	G	0.0127	BTA-77248	96,597,778	A	G	0.0062
ss86310942	96,220,857	A	C	0.0046	ss86340510	96,601,544	A	G	0.0038
ss290490445	96,222,411	A	G	0.0127	ss290490539	96,640,753	G	A	0.0123
ss290490446	96,222,975	G	A	0.0131	ss290490474	96,641,446	A	G	0.0046
ss290490447	96,240,448	C	G	0.0211	rs29022916	96,641,479	A	G	0.0108
ss290490448	96,247,074	A	T	0.0115	ss290490475	96,664,883	A	G	0.0042
ss61557846	96,259,022	A	G	0.0108	ss290490476	96,678,259	G	A	0.0111
ss290490449	96,285,649	C	A	0.0042	ss290490477	96,680,977	G	A	0.0208
ss290490450	96,287,325	A	G	0.0127	rs43479020	96,704,582	A	G	0.0115
ss86324329	96,299,549	A	G	0.0135	BTA-06258	96,723,957	A	G	0.0127
ss290490451	96,304,280	G	A	0.0131	rs29020620	96,724,593	A	G	0.0058
ss290490452	96,306,691	A	G	0.0135	ss290490479	96,762,880	C	A	0.0058
rs29011685	96,314,998	C	G	0.0138	ss290490480	96,782,146	A	G	0.0150
rs43476086	96,322,848	A	G	0.0046	ss290490481	96,786,997	G	A	0.0150
ss290490502	96,337,250	A	G	0.0111	BTA-27374	96,799,041	A	G	0.0146
ss290490453	96,341,752	G	A	0.0127	ss290490482	96,821,525	A	G	0.0065
ss290490454	96,342,307	A	G	0.0042	ss290490483	96,821,967	C	A	0.0150
ss290490455	96,342,553	A	G	0.0042	ss290490484	96,821,975	G	A	0.0150
rs43476044	96,344,643	A	G	0.0042	ss290490485	96,831,027	A	G	0.0150
ss290490456	96,359,318	G	A	0.0027	rs42790107	96,844,636	A	G	0.0154
ss117968766	96,394,191	A	G	0.0104	ss290490486	96,861,052	A	C	0.0135

SNP	Position (bp)	A1	A2	Missing%
ss290490487	96,861,166	G	A	0.0631
ss290490488	96,861,667	G	A	0.1180
ss290490489	96,861,779	G	A	0.0631
ss290490490	96,876,675	C	G	0.0631
ss290490491	96,881,771	G	A	0.0654
ss61518585	96,884,950	A	G	0.0654
ss117968707	96,907,794	A	G	0.0627
ss61540596	96,979,904	A	C	0.0069
