# Supplementary material for: Molecular characterization of a long range haplotype affecting protein yield and mastitis susceptibility in Norwegian Red cattle
Source: BMC Genet. 2011 Aug 11;12:70. doi: 10.1186/1471-2156-12-70 (PMC3171720; doi:10.1186/1471-2156-12-70)

Additional file 2

Figure A1 - Linkage disequilibrium

Pairwise linkage disequilibrium (LD) between all pairs of the 556 single nucleotide polymorphisms (SNPs) genotyped in this study presented by *Bos taurus* chromosome 6 (BTA6) positions (complete LD is indicated in white).

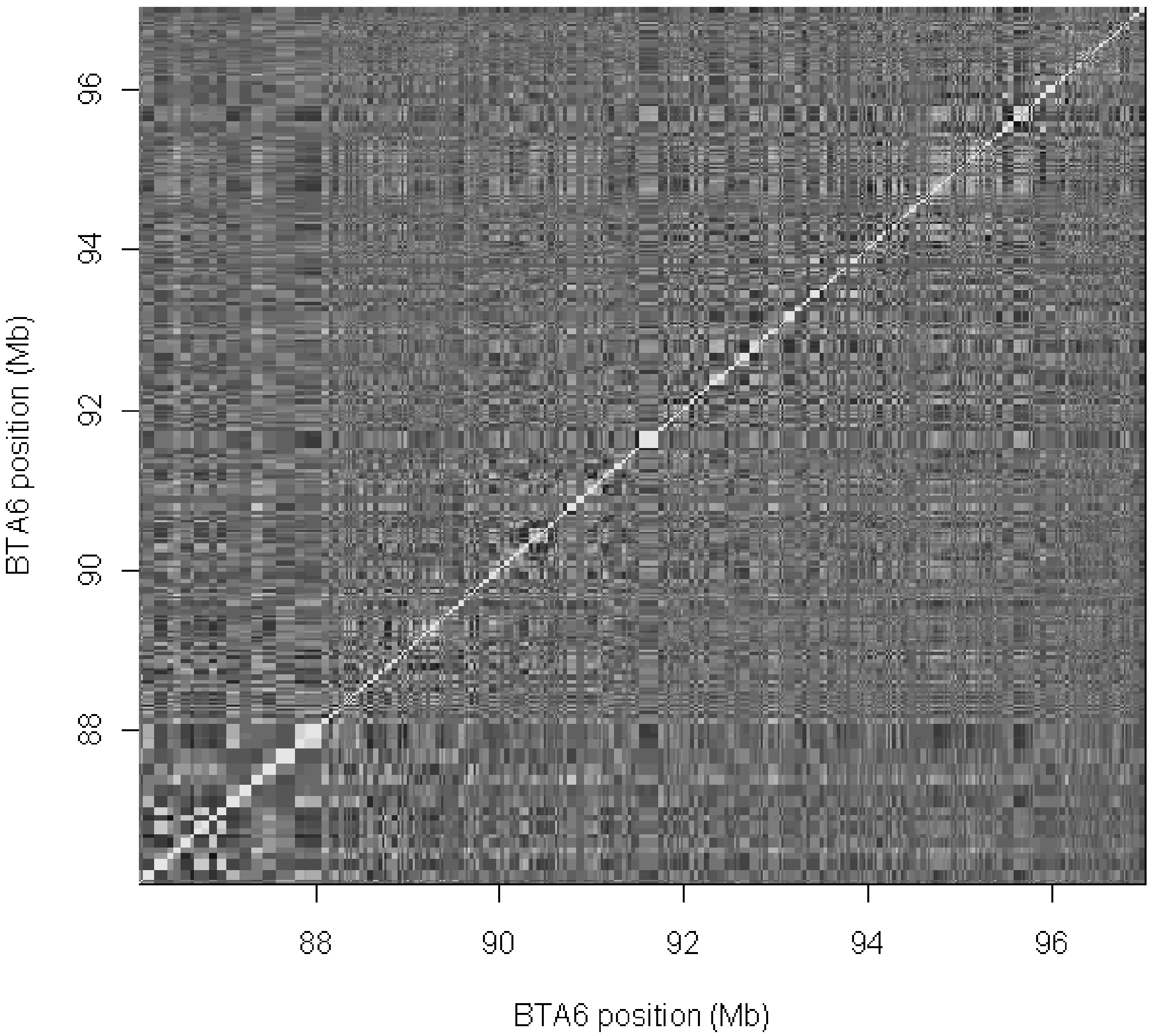

Supplement: Additional file 2 — Linkage disequilibrium. Pairwise linkage disequilibrium (LD) between all pairs of the 556 single nucleotide polymorphisms (SNPs) genotyped in this study presented by Bos taurus chromosome 6 (BTA6) positions (complete LD is indicated in white). [file 1471-2156-12-70-S2.PDF]
